# Sexual Contact as Risk Factor for *Campylobacter* Infection, Denmark

**DOI:** 10.3201/eid2704.202337

**Published:** 2021-04

**Authors:** Katrin Gaardbo Kuhn, Anne Kathrine Hvass, Annette Hartvig Christiansen, Steen Ethelberg, Susan Alice Cowan

**Affiliations:** University of Oklahoma Health Sciences Center, Oklahoma City, Oklahoma, USA (K.G. Kuhn);; Statens Serum Institut, Copenhagen, Denmark (K.G. Kuhn, A.K. Hvass, A.H. Christiansen, S. Ethelberg, S.A. Cowan);; University of Copenhagen Department of Public Health, Copenhagen (S. Ethelberg)

**Keywords:** bacteria, Campylobacter, campylobacteriosis, Denmark, enteric infections, epidemiology, foodborne diseases, food safety, Guillain-Barré syndrome, MSM, risk factors, Salmonella, sexual contact, sexually transmitted infections, Shigella, shigellosis, zoonoses

## Abstract

Campylobacteriosis is a disease of worldwide importance, but aspects of its transmission dynamics, particularly risk factors, are still poorly understood. We used data from a matched case-control study of 4,269 men who have sex with men (MSM) and 26,215 controls, combined with national surveillance data on *Campylobacter* spp., *Salmonella* spp., and *Shigella* spp., to calculate matched odds ratios (mORs) for infection among MSM and controls. MSM had higher odds of *Campylobacter* (mOR 14, 95% CI 10–21) and *Shigella* (mOR 74, 95% CI 27–203) infections, but not *Salmonella* (mOR 0.2, 95% CI 0–13), and were less likely than controls to have acquired *Campylobacter* infection abroad (χ^2^ = 21; p<0.001). Our results confirm that sexual contact is a risk factor for campylobacteriosis and also suggest explanations for unique features of *Campylobacter* epidemiology. These findings provide a baseline for updating infection risk guidelines to the general population.

Foodborne diseases are a global cause of illness and death, imposing an economic burden on not only the food industry but also the public health sector. *Campylobacter* is the most frequently reported gastrointestinal bacterial pathogen in high-income countries (*1*), responsible for an estimated 166 million diarrheal illnesses worldwide and 3.7 million disability-adjusted life years (*2*). The disease is usually self-limiting, with symptoms manifesting as acute watery or bloody diarrhea; treatment is only required for severe cases. *Campylobacter* infection is a causal factor for Guillain-Barré syndrome, a peripheral nerve disorder, which can potentially cause paralysis. Incidence of *Campylobacter* infection is higher in men and boys than in women and girls, and several countries report high incidence in children <5 years of age and in young adults (*1*,*3*). In most high-income countries, infection with *Campylobacter* is notifiable as part of national surveillance programs for infectious diseases. 

Campylobacteriosis is a zoonotic disease; poultry, wild birds, pets, and farm animals are the main reservoirs. Transmission to humans occurs primarily through unsafe handling or consumption of raw or undercooked chicken, consumption of raw milk, or contact with domestic animals (*4*–*6*). However, a large proportion of cases cannot be easily explained by these factors and it has been suggested that other infection routes (e.g., the environment) are equally important in explaining transmission of this disease (*4*,*7*). Some zoonotic pathogens such as *Shigella* spp., *Giardia lamblia*, and *Entamoeba histolytica* have been associated with high risk for infection among men who have sex with men (MSM) because of anal–oral contact (*8*–*12*). Even though several outbreaks have been reported and observational studies have described a high incidence of *Campylobacter* infection among MSM (*8*,*13*,*14*,*15**–**21*), sexual contact is not officially considered among its risk factors for MSM or heterosexual partners in general. However, the transmission potential and incidence of gastrointestinal illnesses among MSM and heterosexual partners engaging in anal–oral sexual contact is difficult to evaluate based on laboratory data only, which does not contain sexual exposure information. 

In Denmark, some infectious diseases, including all foodborne and most sexually transmitted infections, must be reported to Statens Serum Institut (SSI; https://en.ssi.dk), the national institute for infectious diseases of Denmark, through the national surveillance and notification system. The surveillance system comprises 2 parts: clinical notifications and laboratory notifications. Clinical notifications cover diseases that must be reported (Appendix), including serious infectious diseases (e.g., meningitis and tuberculosis), sexually transmitted diseases, and *Shigella* spp. infections. These notifications include relevant patient information, primarily the unique individual Civil Registration System (CPR) number (*22*) and circumstances possibly affecting transmission of the infection, such as sexual contact, foreign travel, and contact with hospitals. General practitioners and hospital physicians fill out details on paper forms that are sent to SSI for manual entry into the database of the national clinical reporting system for infectious diseases. While MSM sexual contact is listed as a possible factor related to transmission, notifications do not include information on anal-oral contact between heterosexual partners. 

The laboratory notification system receives reports of all gastrointestinal infections from certain microorganisms, including campylobacteriosis, salmonellosis, and shigellosis, for which clinical microbiological laboratories are obliged to report findings; reports also include the patient’s CPR number. Laboratory notifications can include information on travel but this is not mandatory. Notifications of gastrointestinal infections are registered and stored in the Denmark Register of Enteric Pathogens. By using each patient’s unique CPR number, duplication of patient records can be avoided and multiple reports for individual patients from the clinical infectious disease and enteric infections databases can be coupled. These data can then be used to generate linked datasets of notifiable diseases and possible explanatory or risk factors. We used these high-quality national surveillance data in an individually matched case–control study undertaken to investigate the frequency of *Campylobacter* infections among MSM in Denmark. 

## Methods

### Study Design and Participants

**Figure 1:**
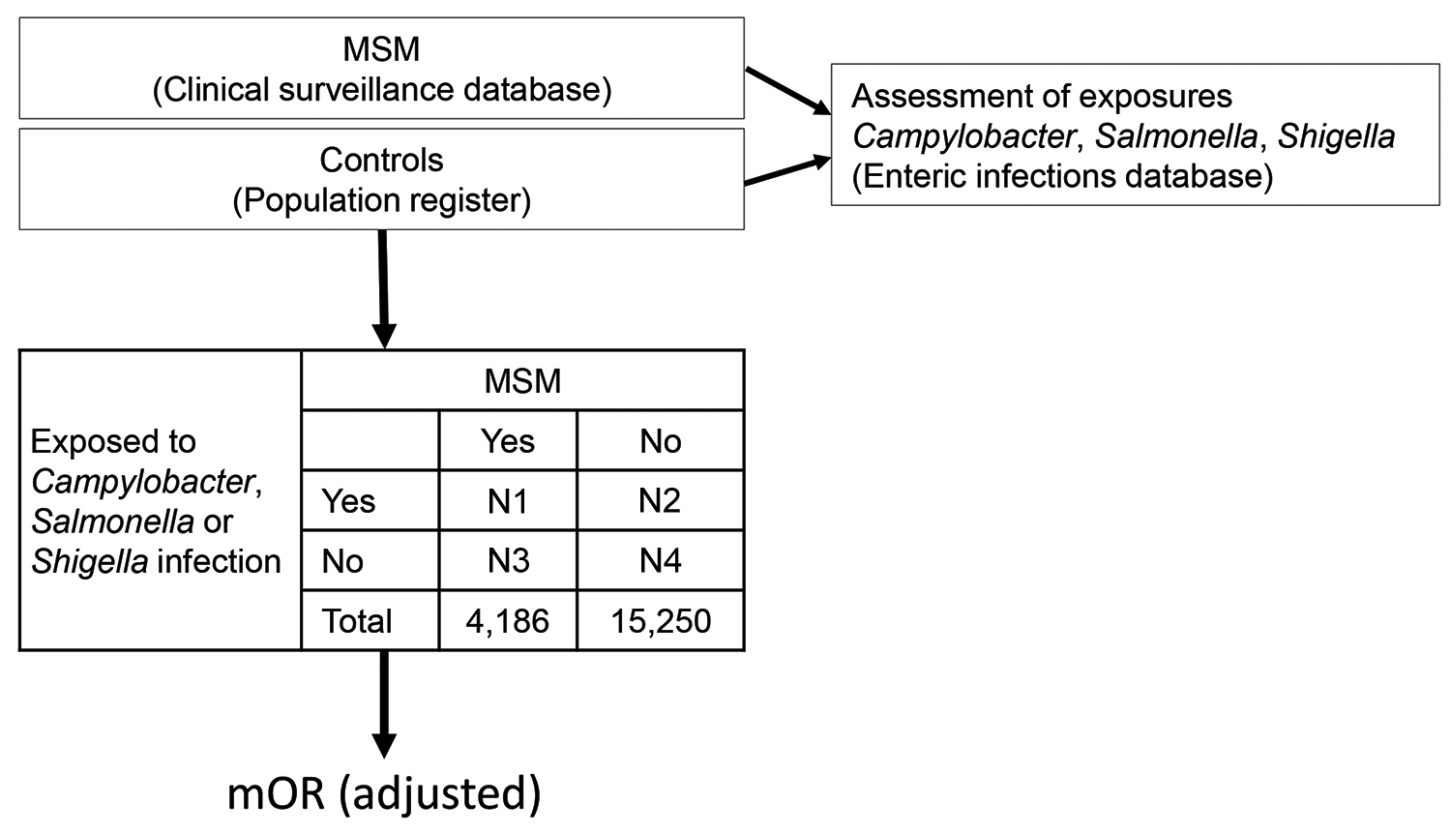
Inverted case-control study design in study of sexual contact as risk factor for *Campylobacter* infection, Denmark, 2010–2018. mOR adjusted for foreign travel, year of notification, infection with any of the other pathogens, and recurrent infections. mOR, matched odds ratio; MSM, men who have sex with men.

We undertook a national retrospective, individually matched case-control study, with a 9-year study period, 2010–2018, among men >18 years of age residing in Denmark. Using an inverted case-control design, we considered MSM as case-patients and infections with *Campylobacter* spp., *Shigella* spp., or *Salmonella* spp. as exposures (Figure 1). We defined an MSM case-patient as a man >18 years of age, with >1 notification of any infectious disease (Appendix) acquired through MSM contact reported to SSI in a clinical notification during the study period. However, for men identified by CPR number in >1 notification during the study period, we included data from only 1 report. We excluded those <18 years of age at the time of the notification or with an incomplete CPR number. For the control group, we randomly selected men >18 years of age from CPR digital records. We individually matched each MSM case-patient to (ideally) 3–5 controls by age (by year and month of birth) and municipality of residence. To determine exposures, we drew information on laboratory-confirmed infections with *Campylobacter* spp., nontyphoidal *Salmonella* spp., and *Shigella* spp. from the enteric infections database. 

### Extraction of Data

From the clinical infectious disease database, we extracted clinical notifications of men with an infectious disease acquired through MSM contact during 2010–2018, and from the enteric infections database, laboratory notifications of *Campylobacter* spp., *Salmonella* spp., and *Shigella* spp. infections over the same period. For each disease notification, we obtained the CPR number of the MSM case-patient, year of notification, and whether the infection was assumed to have been acquired in Denmark or abroad. We included notifications for all species of the 3 pathogens. Data on campylobacteriosis, salmonellosis, and shigellosis were extracted for both sexes and all age groups but only reports for men >18 years of age at the time of notification were included in the study. For calculation of age-specific incidence of the pathogens, we used national population data available from Statistics Denmark (https://www.statistikbanken.dk). 

### Calculations and Statistical Analysis 

To illustrate the age distribution of infection, we divided case-patients and controls into 2 groups: those <40 years of age and those >40 years of age. We analyzed the difference between the frequency of travel-acquired and recurrent infections in the MSM and control groups, age distributions of infections, and the geographic distribution of MSM case-patients in comparison to the general population using χ^2^ tests. We calculated unadjusted and adjusted matched odds ratios (mORs) with 95% CIs for the 3 exposures, *Campylobacter*, *Salmonella*, and *Shigella* infections, in MSM case-patients and controls using conditional logistic regression. The analysis was adjusted for foreign travel, year of notification, infection with any of the other pathogens included in the study, and recurrent infections. We analyzed the distribution of MSM notifications and gastrointestinal diseases over time using a simple regression for trend. We performed all data analyses using Stata version 14 (StataCorp, https://www.stata.com). 

This study was approved under the general agreement for noninterventional database studies between the Danish Data Protection Agency and Statens Serum Institut (reference number 2008-54-0474). According to regulations in Denmark, ethics committee approval is not required for studies that do not involve analysis of biologic material from human subjects.

## Results 

From the clinical infectious disease database, we extracted 4,269 individual reports (only 1 per person) of men who had acquired a notifiable disease through MSM contact during January 1, 2010–December 31, 2018. Of these, 83 (1.9%) men were excluded because they did not have either a valid CPR number or an address in Denmark. For the control group, we extracted 15,250 randomly selected matched male controls from the CPR registry. The mean age was 41 years (median 40 years, range 18–88 years) for both the case-patients and the controls. Case-patients and controls were geographically distributed throughout Denmark, but a significantly larger proportion of the study population than the general population resided in the Capital Region (χ^2^ = 1,400; p<0.0001). 

From the Register of Enteric Pathogens, we extracted 49,321 notifications of infections with >1 of the 3 bacterial pathogens (exposures); 748 patients had registrations for >2 recurrent infections with the same pathogen during the study period (Table 1). Most (76%) notifications were for *Campylobacter* infections: 55% *C. jejuni*, 4% *C. coli*, and 40% other *Campylobacter* spp. with no species reported. In the MSM group, a total of 132 *Campylobacter*, 3 *Salmonella*, and 64 *Shigella* infections were reported during the study period (Table 1). In the control group, we observed 74 *Campylobacter*, 44 *Salmonella*, and 4 *Shigella* infections (Table 1). Compared with controls, a higher proportion of MSM case-patients <40 years of age had *Campylobacter* infections (Table 1), although this difference was not statistically significant (χ^2^ = 3; p = 0.08). 

**Table 1:** Gastrointestinal diseases reported to Statens Serum Institut, Denmark, 2010–2018*

Pathogen	Patients with enteric infection exposures, n = 49,321	MSM, n = 4,186	Controls, n = 15,250
*Campylobacter*	37,602	132 (3)	74 (0.5)
Annual incidence	7.4‡	35§	5.4§
Recurrent infections	524 (1.4)	4 (3)	0
Foreign travel†	7,252 (19)	15 (11)	24 (32)
Age <40 y	19,930 (53)	79 (60)	35 (47)
*Salmonella*	10,450	3 (0.1)	44 (0.3)
Annual incidence	2.1‡	7.2§	3.2§
Recurrent infections	109 (1)	0	0
Foreign travel†	3,916 (37)	1 (33)	9 (20)
Age <40 y	3,380 (32)	1 (33)	15 (34)
*Shigella*	1,269	64 (1.5)	4 (0.03)
Annual incidence	0.2‡	17§	0.3§
Recurrent infections	15 (1.2)	0	0
Foreign travel†	527 (42)	7 (11)	1 (25)
Age <40 y	838 (66)	39 (61)	3 (75)
*Values are no. (%) except as indicated, except incidence, which is given as cases/10,000 population. MSM, men who have sex with men. †Travel information was unknown for 57 patients with *Campylobacter* infections, 25 patients with *Salmonella* infections, and 55 patients with *Shigella* infections (both MSMs and controls). ‡Annual incidence refers to incidence in the population of Denmark. §Annual incidence refers to incidence in the sample population.			

**Figure 2:**
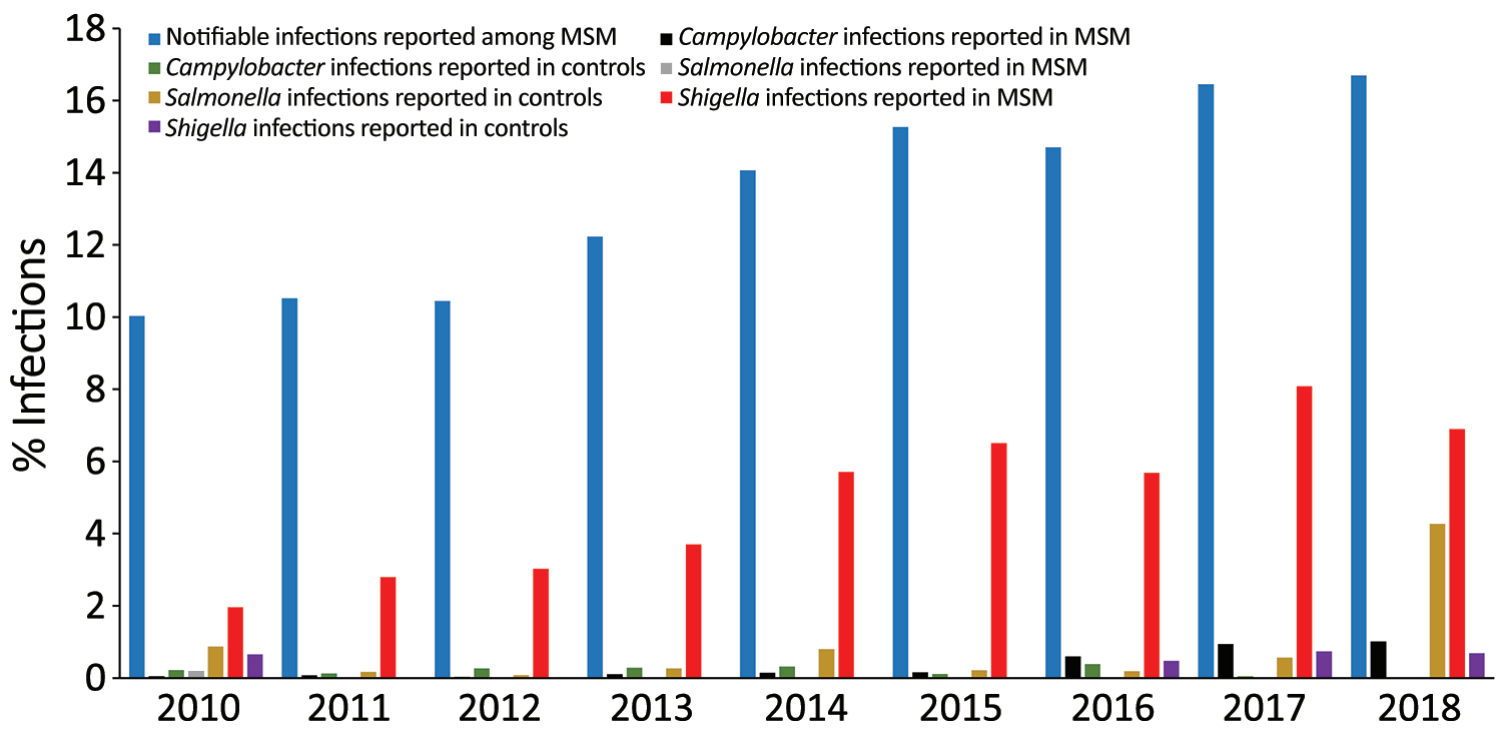
Percentages of clinical notifications of infections acquired through MSM contact (notifiable infections) and *Campylobacter*, *Salmonella*, and *Shigella* infections reported among MSM and controls in study of sexual contact as risk factor for *Campylobacter* infection, Denmark, 2010–2018. MSM were men >18 years of age notified of any infectious disease acquired through sexual contact with another man. Controls were men >18 years randomly selected from the Denmark population register. MSM and controls <18 years of age or who did not have a valid national civil registration number were excluded from the study. MSM, men who have sex with men.

Overall, we found that the odds of a *Campylobacter* infection were 14 times higher among MSM than controls (Table 2). MSM case-patients also had 74 times higher odds than controls of being infected with *Shigella*, a pathogen known to be transmitted by sexual contact (Table 2). However, we found no significant difference between *Salmonella* infection rates in MSM case-patients and controls (Table 2). MSM case-patients who were infected with *Campylobacter* were significantly less likely to have acquired their infection abroad compared with controls (χ^2^ = 21; p<0.001), which was not the case for *Salmonella* or *Shigella*. Over the study period, there were 4 (3%) recurrent *Campylobacter* infections in the MSM group compared with none in the control group (statistical analysis not possible); there were no recurrent *Salmonella* or *Shigella* infections in the MSM or control groups. During the study period, we observed an increase among MSM case-patients in clinical infections acquired through MSM contact and *Campylobacter* or *Shigella* infections (Figure 2, t = 5–11; p<0.001). We did not observe any change in the proportion of *Salmonella* among MSM case-patients (t = −2; p = 0.1) or for any of the 3 pathogens in the control group (Figure 2, t = −1 to 2; p = 0.1–0.4). 

**Table 2:** Matched odds ratios by gastrointestinal infection among MSM and controls in study of sexual contact as risk factor for *Campylobacter* infection, Denmark, 2010–2018*

Pathogen	MSM, no. (%), n = 4,186	Controls, no. (%), n = 15,250	Unadjusted comparison			Adjusted comparison†	
			mOR (95% CI)	p value		mOR (95% CI)	p value
*Campylobacter*	132 (3)	74 (0.5)	16 (11–23)	<0.001		14 (10–21)	<0.001
*Salmonella*	3 (0.07)	5 (0.03)	3 (0.7–13)	0.132		0.2 (0.02–1.3)	0.09
*Shigella*	64 (1.5)	4 (0.03)	105 (37–307)	<0.001		74 (27–203)	<0.001

*mOR, matched odds ratio; MSM, men who have sex with men.  †Adjusted for foreign travel, year of notification/infection, infection with any of the other pathogens and recurrent infections.

## Discussion

We present surveillance data–driven evidence that campylobacteriosis can be transmitted through sexual contact. Among our study group of MSM case-patients, the odds of being infected with *Campylobacter* was 14 times higher than among controls. We observed a similar pattern for shigellosis, which is known to be transmitted by sexual contact, but not for *Salmonella* infection. In Denmark, *Salmonella* infections are considered almost exclusively foodborne whereas domestic *Shigella* infections are regarded as primarily sexually transmitted and secondarily as foodborne or transmitted through general person-to-person contact. *Campylobacter* infection is linked to several different transmission routes (*4*), handling or consuming of poultry long considered the most notable. 

However, our results reinforce information from many reports suggesting *Campylobacter* can be transmitted through sexual contact; these reports provide explanations for unique aspects of *Campylobacter* epidemiology supported by biologic facts from other foodborne bacteria. Reported outbreaks among MSM in Canada (*14*,*19*,*23*,*24*) and previous observations of higher infection rates in homosexual men and HIV-positive patients (*8*,*13*,*17*,*20*,*21*,*25*,*26*) indicate the likelihood that *Campylobacter* can be transmitted through sexual contact. In spite of this, sexual contact has not traditionally been considered a possible transmission route in sporadic campylobacteriosis cases and therefore has been excluded as a possible risk factor in published case-control studies (*4*). We were unable to assess the risk of *Campylobacter* transmission through anal–oral contact between heterosexual partners because only MSM sexual contact is reported on notifications. However, it is highly likely that the risk of infection through this type of sexual contact is equally relevant for heterosexual and MSM partners. 

Several aspects of *Campylobacter* epidemiology remain to be clarified, such as why the disease is more common among men (*3*,*13*,*27*–*29*). Many explanations have been proposed, including differences in food handling and preparation, healthcare-seeking behaviors, and physiologies. We provide an additional explanation: adult men practicing sex with other men are at significantly higher risk (p<0.001) of campylobacteriosis. In this study, *Campylobacter* incidence in the MSM group was almost 5 times higher than in the general population, which could be a partial driver for higher incidence in men. 

Another feature observed in surveillance statistics from several countries was the biphasic age distribution of *Campylobacter* infections, showing pronounced peaks in children <5 years of age and young adults 20–40 years of age (*3*,*30*–*33*). In the 20–40-year age group, these peaks have also been observed for *Shigella* (*34*,*35*), but not for other foodborne bacteria, such as *Salmonella*. These findings have been explained by the secondary weaning phase, meaning that young adults away from home are less aware of proper hygiene practices, secondary infections from caring for young children at home, or hormonal and behavioral factors. When comparing different age groups, we saw higher *Campylobacter* infection rates in MSM <40 years of age compared with controls, although this difference was not statistically significant because of the small sample size. On the basis of our results, we suggest that, in countries with a clear biphasic *Campylobacter* age distribution, some of the high infection frequency among young adults might be explained by sexual transmission among persons in this very sexually active age group. 

Bacterial pathogens can be transmitted through fecal–oral contact during sex, either directly, through anal–oral contact or anal–penile–oral contact, or indirectly, such as through the use of sex toys or fingers. In 1974, sexual contact was recognized as a risk factor for infection with *Shigella* spp. (*11*), and such contact is now widely acknowledged as one of the most important transmission routes for shigellosis. The probability of infection with a foodborne bacterium from fecal–oral contact is directly related to the infectious dose. For *Shigella* spp. this dose can be as low as 10–1,000 organisms, and for *Campylobacter*, 500–10,000 organisms (*36*). The infectious dose for *Salmonella* spp. varies (*37*) but might be as high as 1 million bacterial cells. Pathogens with a low infectious dose are easier to transmit from person to person, including through sexual contact; on the basis of our findings, we tentatively propose that this might be the case for *Shigella* and *Campylobacter*, but not *Salmonella*. 

In Denmark and the rest of Europe, campylobacteriosis and shigellosis are often acquired abroad; 30%–50% of infections are reported as travel related (*3*,*33*). This possibility was reflected in our control group, but *Campylobacter* infections among MSM were significantly less likely to have been travel related (p<0.001). Assuming that many *Campylobacter* and *Shigella* cases in the MSM group have been acquired by sexual contact, this domestic pattern is not surprising because MSM contact is most commonly not a holiday experience and infection is more likely to happen at home. 

During the study period, there was a significant (p<0.001) increase in the proportion of MSM case-patients infected with *Shigella* or *Campylobacter*, but not with *Salmonella*; we found no increase in the control group. Incidence of campylobacteriosis in Denmark has increased recently (*3*), reflecting a combination of actual increases in cases of the disease, changes in diagnostic techniques, and improved electronic notification. During the study period, laboratory diagnosis of gastrointestinal pathogens has transitioned from culturing to more sensitive PCR testing of feces. This change could explain some of the increase in recorded *Shigella* and *Campylobacter* infections; however, similar increases would be expected for *Salmonella* and for the number of infections in the control group. An increasing trend over time would also be observed if there had been larger outbreaks of *Shigella* and *Campylobacter* among MSM case-patients later in the study period, but only 1 outbreak of shigellosis was reported, and none of campylobacteriosis among MSM during this time (*38*). The most likely explanation for the observed increase in *Shigella* and *Campylobacter* among MSM case-patients is the concurrent increase in the proportion of infections transmitted through MSM contact reported to SSI. More extensive reporting has increased the amount of data available in the electronic databases, in turn increasing the likelihood of discovering infections in the MSM group. 

Our study had 2 major strengths: use of routinely collected high-quality national surveillance data for our analyses and results supported by biologic evidence and demographic information, such as the infectious doses and the low level of travel-related infections in the MSM group that explain some of the unique characteristics of *Campylobacter* transmission. The first limitation is that, although clinical notifications of diseases in Denmark can include MSM contact as possibly associated with the circumstances of the infection, such inclusions are based on patient statements, and only HIV, syphilis, gonorrhea, and hepatitis infections must be reported. Therefore, the MSM population group probably did not include all men with an infection acquired through MSM contact during the study period. It is also very likely that the control group included some MSM and that some reported *Campylobacter*, *Shigella*, or *Salmonella* infections in the control group were acquired through MSM contact. However, such an underestimation would tend to lower the estimates of association toward 1 and, in fact, approximate the odds ratios to risk ratios. Differences between case-patients and controls concerning how exposure was determined (i.e., from a notification to SSI about infection with an enteric pathogen) could have affected the results. However, because physicians and laboratories in Denmark operate under the same guidelines and procedures and the notification records are uniformly registered, such differences are unlikely to have had an effect. 

The results in this study provide a measure of the risk associated with a notifiable infectious disease among a specific group of MSM and not among a general MSM population. However, considering the robust mORs and corresponding CIs combined with published reports about high *Campylobacter* incidence in MSM populations, we believe that our findings can be extrapolated to MSM in general. The case-patient group might overrepresent risk-taking persons more likely to practice unsafe sex, and if that is the case, the increased risk for *Campylobacter* infection through sexual contact would primarily apply to this high-risk group. However, it can also be argued that this risk applies to the wider MSM population because condom use would not eliminate the infection risk associated with several fecal–oral transmission routes. The case-patients we selected might also have a high frequency of known risk factors, such as use of proton pump inhibitors, certain occupations, or increased contact with animals, that we could not account for in this study. 

Another limitation of the data used in our study was the incomplete travel information for persons with reported infections. Travel history was unknown for patients with 25% of *Salmonella*, 55% of *Shigella*, and 57% of *Campylobacter* infections because of incomplete registration and lack of follow-up interviews. This proportion was similar between case-patients and controls. In spite of this, our results still indicated that MSM are less likely to have acquired *Campylobacter* or *Shigella* infections abroad. Also, although our data covered all infections reported as acquired through MSM contact and all confirmed cases of campylobacteriosis, salmonellosis, and shigellosis, these do not account for all cases. Notifications are based on passive surveillance and underestimate the true incidence because persons with infections who do not seek medical attention are not captured (*39*). However, because our system enabled linkage of individual data collected, we believe that these results are as accurate as possible and could be generalized to similar settings. Lack of association between MSM contact and *Salmonella* infection might reflect the true situation or that a larger sample size is needed. 

The cycle of transmission through sexual contact among adults contributes substantially to the burden of disease in highly industrialized countries, but likely not in low-middle income settings where limited hygiene and sanitation constitute greater risk factors. Therefore, the external validity of the results in some locations should be considered and we encourage public health agencies in other countries to replicate this study to corroborate the findings. Finally, as we did not directly measure *Campylobacter* infection transmitted through anal-oral contact, the association is speculative and our conclusions are derived from surveillance data analyses in combination with knowledge gained from studies of other bacteria.

Our findings indicate a strong likelihood that *Campylobacter* can be transmitted during sexual contact. Given previous reports of outbreaks and high incidence of *Campylobacter* among MSM, this is not surprising. Combining this theory with high-quality national surveillance data, our results offer additional reasonable explanations for why surveillance statistics from some countries show that adult men are more frequently infected with *Campylobacter* than are women and why *Campylobacter* incidence peaks among young adults. Overall, our findings not only address epidemiologic questions but also highlight a need to inform the public about the risk of infection through sexual activity in regions where *Campylobacter* incidence exhibits patterns similar to those in Denmark. These results are primarily applicable to adults in high-income settings in some countries; further studies are warranted among the general MSM population rather than only those with a reported notifiable disease. However, considering the high burden of campylobacteriosis in Europe, the United States, and Australia, incorporating knowledge from these findings into general information campaigns might encourage the use of precautionary measures during sexual contact, which could, in turn, lead to lower infection rates and reduced overall costs to society. 

Appendix List of notifiable infectious diseases that must be reported through clinical notifications to Statens Serum Institut in Denmark (as of January 2021).
