# Supplementary material for: Sexual Contact as Risk Factor for Campylobacter Infection, Denmark
Source: Emerg Infect Dis. 2021 Apr;27(4):1133–40. doi: 10.3201/eid2704.202337 (PMC8007285; doi:10.3201/eid2704.202337)
Supplement: Appendix — List of notifiable infectious diseases that must be reported through clinical notifications to Statens Serum Institut in Denmark (as of January 2021). [file 20-2337-Techapp-s1.pdf]

Article DOI: https://doi.org/10.3201/eid2704.202337

Sexual Contact as Risk Factor for *Campylobacter* Infection, Denmark

Appendix

List of notifiable infectious diseases that must be reported through clinical notifications to Statens Serum Institut in Denmark (as of January 2021)

AIDS

Anthrax

Botulism

Carbapenemase-producing organisms

Creutzfeldt-Jacobs disease

Diphtheria

Avian influenza in humans

Hepatitis A (acute), B (acute), B (chronic), C (acute), C (chronic)

Hemolytic uremic syndrome

Haemorrhagic fever (Lassa, Marburg, Ebola)

Cholera

Congenital rubella

Smallpox

Legionella pneumonia

Leprosy

Leptospirosis

Meningococcal disease

Morbilli

Neuroborreliosis

Ornithosis (psittacosis)

Pandemic influenza

Paratyphoid

Parotitis

Pertussis in children <2 years of age

Plague

Spotted typhus fever (typhus exanthematicus)

Polio

Purulent meningitis

Rabies

Rubella in pregnancy

Severe Acute Respiratory Syndrome (SARS)

Shigella

Tetanus

Tuberculosis

Typhus

Waterborne diseases

Verotoxin-producing *Escherichia coli* (VTEC)
